# Supplementary material for: The Systems Biology Research Tool: evolvable open-source software
Source: BMC Syst Biol. 2008 Jun 29;2:55. doi: 10.1186/1752-0509-2-55 (PMC2446383; doi:10.1186/1752-0509-2-55)
Supplement: Additional file 1 — SBRT Archive. An archive of the current version of the Systems Biology Research Tool. [file 1752-0509-2-55-S1.zip › sbrt-1.4.0/doc/developers_guide/api/sbrt/shell/text/class-use/PipeSetFormat.html]

Uses of Class sbrt.shell.text.PipeSetFormat


|  |  |  |
| --- | --- | --- |
| **Overview** \| **Package** \| **Class** \| **Use** \| **Tree** \| **Deprecated** \| **Index** \| **Help** | |  |
| PREV   NEXT | **FRAMES**    **NO FRAMES**     **All Classes** |  |


---


## **Uses of Class sbrt.shell.text.PipeSetFormat**

| Packages that use PipeSetFormat | |
| --- | --- |
| **sbrt.shell.text** | Provides classes and interfaces for defining formats for basic objects. |
| **sbrt.shell.text.fba** | Provides classes and interfaces for defining formats for objects relevant to Flux Balance Analysis. |

Uses of PipeSetFormat in sbrt.shell.text

| Methods in sbrt.shell.text that return PipeSetFormat | |
| --- | --- |
| `static PipeSetFormat<java.lang.String>` | `PipeSetFormat.getBasic()`             Returns a `PipeSetFormat` that does not format or parse the elements supplied to it. |
| `PipeSetFormat<K>` | `EqualsMapFormatV1.getSetFormat()`             Returns the format used for sets of variables. |
| `PipeSetFormat<java.lang.String>` | `AppInputFileLineFormatVersion1.getSetFormat()`             Returns the format used for sets of variables. |
| `PipeSetFormat<K>` | `ColonMapFormatV1.getSetFormat()`             Returns the format used for sets of variables. |

Uses of PipeSetFormat in sbrt.shell.text.fba

| Methods in sbrt.shell.text.fba that return PipeSetFormat | |
| --- | --- |
| `PipeSetFormat<java.lang.String>` | `FbaOptHeaderParserV1.getPipeSetFormat()`             Returns the format used for sets of strings. |
| `PipeSetFormat<java.lang.String>` | `FbaOptHeaderFormatterV1.getPipeSetFormat()`             Returns the format used for sets of strings. |
| `PipeSetFormat<java.lang.String>` | `FluxCapFormatV1.getSetFormat()`             Returns the format used for sets of variables. |
| `PipeSetFormat<java.lang.String>` | `ConstraintsFileLineFormatV1.getSetFormat()`             Returns the format used for sets of variables. |
| `PipeSetFormat<java.lang.String>` | `FluxVectorFormatV1.getSetFormat()`             Returns the format used for sets of variables. |
| `PipeSetFormat<java.lang.String>` | `CatalystSetFormatV1.getSetFormat()`             Returns the format used for sets of catalyst names. |
| `PipeSetFormat<java.lang.String>` | `IrfFormatV1.getSetFormat()`             Returns the format used for sets of reaction names. |
| `PipeSetFormat<LinearComb<java.lang.String>>` | `ConstraintsFileLineFormatV2.getSetFormat()`             Returns the format used for sets of variables. |
| `PipeSetFormat<java.lang.String>` | `FluxomeSolFileLineFormatV1.getSetFormat()`             Returns the format used for sets of variables. |
| `PipeSetFormat<java.lang.String>` | `RxnNameSetFormatV1.getSetFormat()`             Returns the format used for sets of reaction names. |

---


|  |  |  |
| --- | --- | --- |
| **Overview** \| **Package** \| **Class** \| **Use** \| **Tree** \| **Deprecated** \| **Index** \| **Help** | |  |
| PREV   NEXT | **FRAMES**    **NO FRAMES**     **All Classes** |  |


---
